# Angiogenic efficacy of Heparin on chick chorioallantoic membrane

**DOI:** 10.1186/2045-824X-4-8

**Published:** 2012-04-18

**Authors:** Reji Bhuvanendran Rema, Karthick Rajendran, Malathi Ragunathan

**Affiliations:** 1 Department of Genetics, Dr. ALM PG IBMS, University of Madras, Taramani Campus, Chennai, 600 113, Tamil Nadu, India

**Keywords:** Heparin, Angiogenesis, Chorioallantoic Membrane (CAM), Endothelial cells (ECs)

## Abstract

Heparin is an anticoagulant agent known to have diverse effects on angiogenesis with some reports suggesting that it can induce angiogenesis while a few have indicated of its inhibitory property. Cancer patients treated for venous thromboembolism with low molecular heparin had a better survival than the unfractionated heparin (UFH). Heparin is known to interact with various angiogenic growth factors based on its sulfation modifications within the glycosaminoglycan chains. Therefore it is important to study the mechanism of action of heparin of different molecular weight to understand its angiogenic property. In this concern, we examined the angiogenic response of higher molecular weight Heparin (15 kDa) of different concentrations using late CAM assay. Growth of blood vessels in terms of their length and size was measured and thickness of the CAM was calculated morphometrically. The observed increase in the thickness of the CAM is suggestive of the formation of capillary like structures at the treated region. Analysis of the diffusion pattern showed internalized action of heparin that could affect gene expression leading to proliferation of endothelial cells. Angiogenesis refers to formation of new blood vessels from the existing ones and occurrence of new blood vessels at the treated area strongly confirms that heparin of 15 kDa molecular weight has the ability to induce angiogenesis on CAM vascular bed in a dose dependent manner. The results demonstrate the affinity of heparin to induce angiogenesis and provide a novel mechanism by which heparin could be used in therapeutics such as in wound healing process.

## Background

Heparin, a member of the glycosaminoglycan family of carbohydrates found on the cell surface and in the extracellular matrix [1] has the potential to induce angiogenesis [2,3]. It was shown to bind both with angiogenic [4] and anti-angiogenic proteins favoring and stabilizing the association with their respective receptors [5]. Heparin present at the basal site of blood vessels act as matrix receptors by interacting with a variety of basement membrane proteins and is involved in the binding and internalization of lipoprotein lipase on endothelium [6]. Heparin present in the extra cellular matrix (ECM) may act as a reservoir for angiogenic growth factors and will sustain the long-term stimulation of endothelial cells [7]. It was already shown that heparin of different molecular weight could promote angiogenesis [8] on the chorioallantoic membrane of the chick embryo and introduction of heparin directly into the allantoic sac of CAM [9] resulted in dilated sinuous arterial and venous branches suggesting that heparin could induce new blood vessel formation. Poly-l-lysine/heparin complexes were found to stimulate angiogenesis in chick embryo chorioallantoic membrane [10].

We analyzed the angiogenic effect of Heparin (15 kDa) by treating CAM with 3 different concentrations (50, 100 and 150 μM) using gelatin sponges after 72hours of incubation. Biotinylated Heparin contains 5–8 mole-percent biotin and is attached through a 12-atom spacer arm containing a cleavable disulfide group. This has been used mainly to study its localization on CAM using streptavidin labeled with HRP.

Diffusion of Heparin through various layers of CAM and also the morphological changes induced on CAM during treatment was analysed. Thickness of the CAM was also measured to further substantiate the angiogenic response and formation of new blood vessels. *Angioquant* software was used to measure the growth of the vessels by modifying the macroscopic images taken during treatment period. This software is specially made for quantifying angiogenesis using *in vitro* assays and has been employed for CAM with some modifications**.** The results obtained using morphometry and immunostaining of 15 kDa heparin might be of therapeutic significance in angiogenic disorders that requires neovascularisation.

## Materials and Methods

### Materials

Fertilized White leghorn chicken eggs (*gallus gallus*) were purchased from, Tamil Nadu Poultry Research Station, Madras Veterinary University, Nandanam, Chennai, India. Gelatin sponges were from Jhonson & Jhonson Pvt Ltd, Heparin from CALBIOCHEM, USA (Product No. 375054), HRP conjugated streptavidin (HRP-anti-rabbit secondary) from Bangalore Genei, India, Phosphate buffered solution and other common reagents were purchased from Sigma, Aldrich, USA.

### Methods

### The in ovo CAM model

Fertilized chicken eggs weighing 50 ± 2gm were incubated at 37°C in a humidified atmosphere (>60% relative humidity) based on protocol for the Hen's Egg Test-Chorioallantoic Membrane (HET-CAM) method. At day 3 of post incubation, 2 to3 ml of albumin was withdrawn, using a 21-gauge needle, through the large blunt edge of the egg in order to minimize adhesion of the shell membrane with CAM. A square window of 1 cm² was opened in the egg shell and sealed with paraffin film to prevent dehydration and the eggs were returned for incubation. On day 9^th^, gelatin sponges of size of 1 mm³ were placed on the top of growing CAM under sterile condition [11]. The sponges were soaked with 10 μl of 50, 100 and 150 μM concentrations of heparin. Control CAM was treated with 10 μl of 1X PBS. The window was closed with a transparent adhesive tape and the eggs were returned for further incubation till day 12 (72hours) at which vascularization potential of the CAM reaches its maximum. The experimental groups were divided into 4 with each containing 40 numbers. Group1 represents 1X PBS (control), group 2, 3 and 4 corresponds to 10 μl of 50 μM, 100 μM and 150 μM of heparin. Treated CAM was photographed at 0, 24, 48 and 72hours using Cannon digital camera and the images were analyzed with *Angioquant* Toolbox, MATLAB 6.5 software to measure total length and size of blood vessels in micrometer at the area of treatment [12-14].

### Histology

CAM treated with heparin was flooded with Bouin's fixative solution and the treated area was removed, dehydrated and embedded in paraffin wax. Vertical cross sections of 7 μm in thickness were taken using Rotary Microtome (Weswicox, Japan) [15]. The histological sections were observed under Light Microscope at 40X magnification for qualitative assessment and images were taken using Nikon Camera attached with light microscope at 10X magnification.

### Morphometric analysis

Thickness of the CAM was measured from haematoxylin and eosin stained sections with a calibrated objective at 40X magnification using 10X10 calibrated grids in the 10X ocular. Each CAM was measured at 6 different locations from 6 serial cross sections of the same sample. To calculate mean tissue thickness (D*CAM*) all of the representative sections from the particular tissue sample were measured in micrometer and averaged to yield a mean CAM thickness [15].

### Immunohistochemical analysis

Sections of CAM of 7 μM thickness were obtained using microtome after treatment with heparin. After 12hours of incubation at 37°C the sections were dewaxed. Sections were washed with 1X PBS and incubated with HRP conjugated streptavidin (HRP-anti-rabbit secondary) of 1:40 dilution for 1 hour at 37°C. After counterstaining with haematoxylin and eosin dehydration was performed with alcohol. Images were recorded after mounting with 90% of glycerol using Nikon camera attached with light microscope at 40X magnification. Immunohistochemical staining was performed on paraffin-embedded tissue sections with slight modifications**.**

### Data analysis and statistics

All the experiments were performed in triplicate (*n* = 3) unless otherwise specified. Data are presented as mean ± SEM and were analyzed by One-Way ANOVA analysis of variance test, Student’s *t*-test and Tukey post hoc tests as appropriate by Sigma Stat 2.0. p = < 0.001 and p = 0.001 were considered as statistically significant.

## Results

### Visualization of blood vessels on CAM

**Figure 1:**
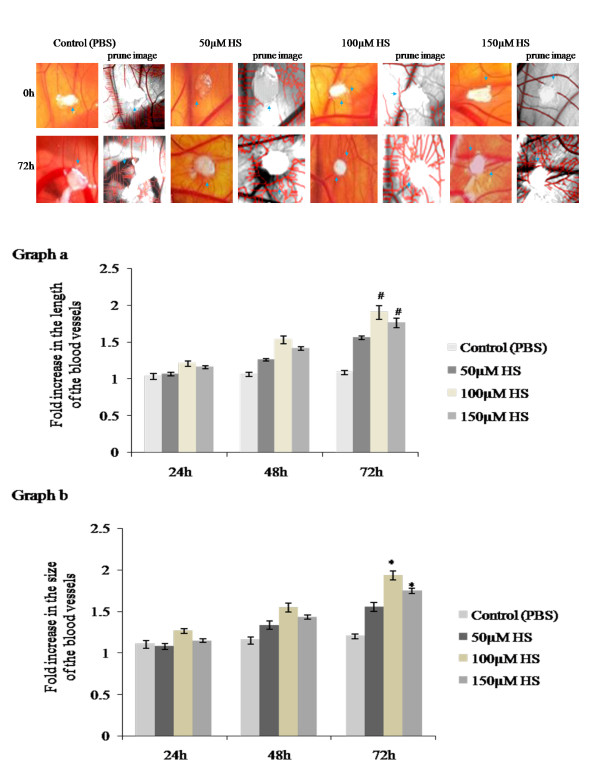
**a. Effect of biotin conjugated heparin on late CAM.** Images of late CAM treated with 50, 100 and 150 μM heparin. Control CAM was treated with 1X PBS. CAM treated with 100 μM heparin shows increased vascularization around the sponge as spoke wheel pattern which is comparatively higher than other concentrations and is also supported by skeletonized prune images by *Angioquant* software. Images are taken using Canon digital camera at 4X magnification and are the result of 3 different set of experiments. Arrow indicates the presence of blood vessels. **1b**. Effect of biotin conjugated heparin on the growth of blood vessels on late CAM. Total length and size of the blood vessels was measured using *Angioquant* software after treating CAM with 50, 100 and 150 μM heparin. Control CAM was treated with 1X PBS. Images recorded at 0, 24, 48 and 72hours were analyzed individually. Values at 0hour were taken as one for all. CAM treated with 100 and 150 μM heparin shows significant increase in vessels length (graph a) and size (graph b) after 72hours of treatment when compared to control. Though both the concentrations shows significant changes, 100 μM heparin shows comparatively higher angiogenic potential. Experiments were performed in triplicate and data presented as mean ± SEM, #p = 0.001 and *p = <0.001 versus control.

We studied the role of heparin in the angiogenic process by performing a CAM assay. An increase in the number of blood vessels was observed for all 3 concentrations (50,100,150 μM) of heparin at 72hours of treatment. Among three concentrations 100 μM heparin showed distinct spoke wheel pattern of vessels with numerous allantoic vessels around the gelatin sponge demonstrating the angiogenic potential of heparin while control CAM treated with PBS showed slow and steady growth of allantoic vessels (Figure 1a). The observation is also supported by the skeletonized prune images obtained from *Angioquant* software (Figure 1a). Angiogenic potential of heparin was further analysed by measuring the growth of the vessels with respect to their length and size and confirmed at various time points of incubation namely at 24, 48 and 72hours of treatment (Figure 1b). A significant increase in the vessel length (#p = 0.001) and size (*p = <0.001) was observed on CAM treated with 100 and 150 μM concentrations of heparin with 100 μM exhibiting maximum angiogenic response.

### Qualitative assessment of angiogenic potential

**Figure 2:**
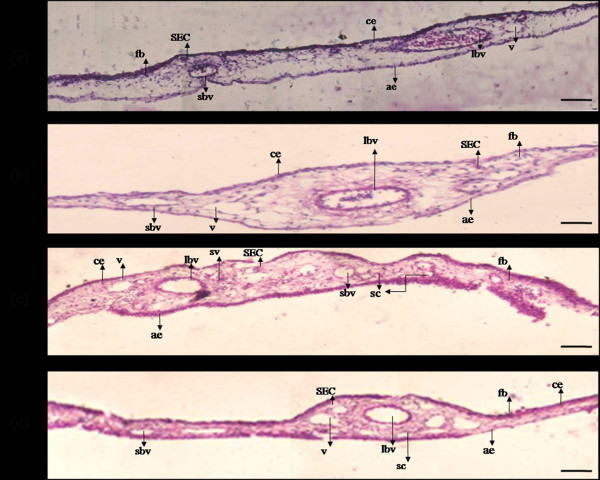
**Effect of heparin on CAM morphology.** (**a**) CAM treated with 1X PBS shows thin chorionic (ce) and allantoic (ae) epithelial layer due to minimal accumulation of fibroblast cells (fb) with subepithelial capillary network (SEC) along with the presence of large blood vessels (lbv) and vein (v). (**b**) CAM treated with 50 μM heparin shows increased thickness at ce and ae due to fb accumulation along with the presence of small blood vessels (sbv) at stroma region. (**c**) CAM treated with 100 μM heparin shows increased thickness at the ae and ce due to increased accumulation of fb with densely packed small blood vessels beneath SEC and also ECs capillary sprouting as small sprouting vessels (sv) which extending towards SEC. Presence of numerous small capillaries (sc) and lbv along with v are also visible at the stroma region. (**d**)150 μM heparin shows diminished fibroblast accumulation at thin stratum and also reiteration of radial arrangement of fb, lbv and sbv are also visible with minimal sprouting of sc. Gelatin sponges did not cause wound or inflammation. Representative tissues are 7 μm in thickness stained with haematoxylin and eosin and images were taken at 40X magnification and are the result of 3 different set of experiments. Bar is 50 μm.

We further demonstrated the angiogenic potential of heparin by studying the morphological features of CAM after treatment (Figure 2). CAM treated with PBS showed uniform tissue thickness without any bulging appearance. Large blood vessel was seen without the appearance of sprouts. The histological appearance was same throughout the chorionic and allantoic epithelial layers. CAM treated with 100 μM heparin showed increased thickness at the thin stratum due to altered fibroblast, epithelial and endothelial cellular morphology. Large numbers of elongated ECs were visible with sprouting appearance. The thickness of the primary stratum also increased due to accumulation of fibroblast at sub-epithelial capillary network (SEC) giving an irregular appearance. Very small capillaries were visible at the connective tissue area due to occasional sprouting from pre existing vessels which tends to pass to the chorionic sinus and could be concluded as the sprouting angiogenic effect at this particular concentration. CAM treated with 50 μM and 150 μM heparin showed irregular appearance of thin stratum at chorionic epithelial layer due to stratification of the cells mainly by fibroblast cells at SEC. A few small capillaries were also visible for 150 μm but radial pattern of fibroblast was reiterated.

### Measurement of CAM thickness (*D*CAM)

**Figure 3:**
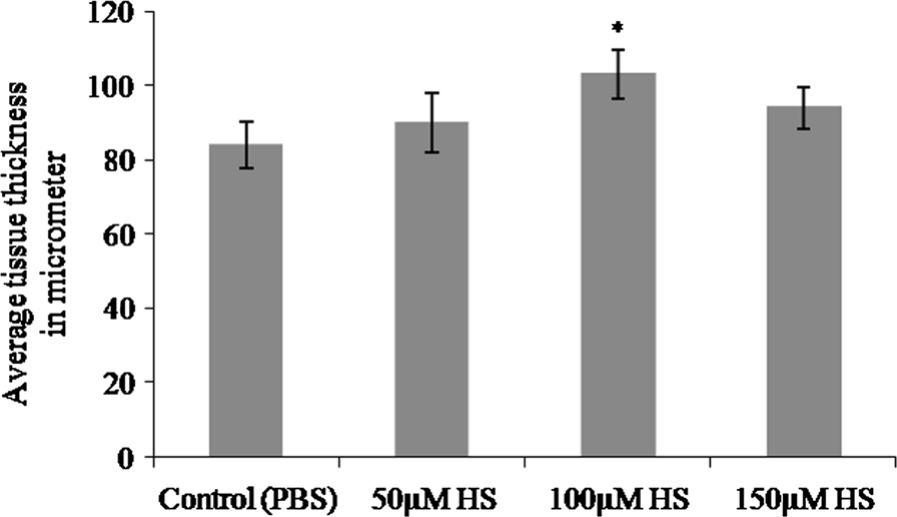
**Thickness of CAM treated with heparin.** Total thickness of the tissue at the area of treatment was measured from haematoxylin and eosin stained paraffin embedded CAM tissues and plotted. Thickness of the CAM treated with heparin is higher than that of control CAM for 72hours. CAM treated with 100 μM heparin showed a significant increase in tissue thickness when compared with the other concentrations.Each value is expressed as mean ± SEM (*p = <0.001 versus control, n = 6).

Growth of the vessels was further confirmed by measuring the thickness of CAM from paraffin embedded vertical sections and plotted as shown in Figure 3. In paraffin-embedded tissue, material shrinkage could be estimated to be ~25% relative to the fresh material and can be assumed to be the same since all tissues were prepared similarly [16]. Thus, shrinkage corrections were not necessary for comparisons of tissue thickness. CAM treated with 100 μM heparin showed significant increase (*p = <0.001) in tissue thickness when compared to the values obtained for 50 μM and 150 μM concentrations.

### Diffusion pattern of heparin

**Figure 4:**
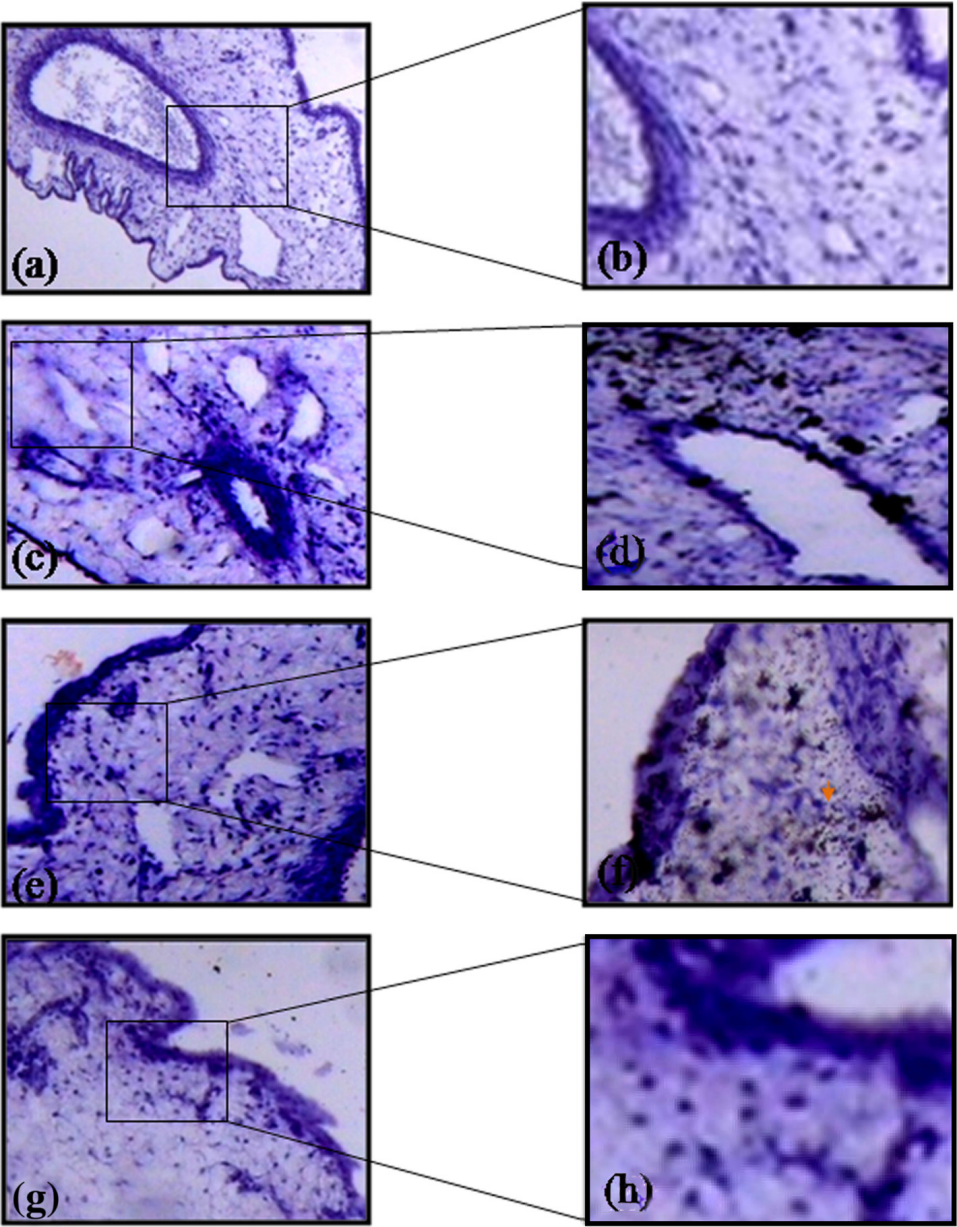
**Diffusion pattern of heparin through various layer of CAM.** CAM treated 1X PBS doesn’t show any diffusion pattern (**a**) stained without HRP and (**b**) with HRP. CAM treated with 50 μM heparin shows diffusion at chorionic (ce) and sub epithelial layers (**c**) stained without HRP and (**d**) with HRP. CAM treated with 100 μM heparin shows diffusion at ce and sub epithelial layers and also at stroma region (**e**) stained without HRP and (**f**) with HRP. CAM treated with 150 μM heparin shows higher intensity of HRP staining due to higher concentration and shows diffusion pattern at ce and sub epithelial layers and also at stroma region (**g**) stained without HRP and (**h**) with HRP. Arrow indicates the presence of biotin-streptavidin complex. Magnification for a, c, e and g is 10X and for b, d, f and h is 40X and are the results of 3 different set of experiments. For a, c, e and g bar is 50 μm and for b, d, f and h bar is 100 μm.

Diffusion of biotin conjugated heparin through various membranes of CAM was analyzed based on its binding with streptavidin. The presence of biotin-strepatavidin complexes is indicative of the diffusion of heparin (Figure 4) through the membrane and the intensity increased with increase in the concentrations. CAM treated with 50 μM heparin showed that it had diffused through the chorionic and sub epithelial cell layers and could favor the activation of fibroblast cell. CAM treated with 100 μM heparin showed that the diffusion occurred not only at the chorionic and sub epithelial layer but also at the stroma in which are present the capillary plexes/blood sinus. This could favor endothelial cell migration which is considered to be the most important step in angiogenesis. CAM treated with 150 μM heparin showed that the diffusion is greater at the sub epithelial layer and in stroma region suggesting that heparin (15 KDA) could favor angiogenesis.

## Discussion

The present study reveals the mechanisms by which heparin of 15 kDa can induce angiogenesis in vitro. New blood vessel formation and its growth on CAM treated with heparin of varying concentrations implied that it has the potential to induce sprouting angiogenesis as observed in the increase in vessel length and size. The usage of *Angioquant* software to measure vessel growth *in vivo* is already reported by others. In sprouting angiogenesis activated Endothelial cells (ECs) favor binding of growth factors such as Vascular Endothelial Growth Factor (VEGF) and basic Fibroblast Growth Factor 2 (FGF2) with its specific receptors which could be activated by various external and internal stimulus [17] resulting in the degradation of extra cellular matrix by Matrix Metalloproteases (MMPs) and proliferation of ECs to form immature blood vessels [18]. These immature blood vessels are further stabilized by the recruitment of mural cells which can contribute to the increase in the size of the vessels [19]. It is understood that heparin could interact with these angiogenic growth factors [20,21] and act as a mitogen for capillary ECs both *in vivo*[22] and *in vitro*[23]. Thus it could be concluded that heparin could induce sprouting angiogenesis on CAM and the growth pattern recorded at different time points showed that its action is dose-dependent with maximum efficacy at 100 μM after 72 hours of treatment.

It was proved that histology of the CAM changes as a response towards the biological materials placed on it including EC migration [24], fibroblast and mast cell accumulation [25,26], changes in thickness of the epithelial layer, increase in the number of capillaries and capillary cords formation [15], presence of apoptotic ECs [27-30]. In the present study heparin is found to alter the morphology of those cells which have a direct role in angiogenesis similar to that reported earlier [15]. The histological changes associated with 100 μM is suggestive of favo-ring blood vessel formation stronger than other two concentrations.

To calculate tissue thickness we measured the distance between chorionic and allantoic layers (in micrometer) that contains different kind of cells and any morphological change found in these cells could affect the entire structure of the CAM. An earlier study showed that angiogenic activity of the substance could be measured by calculating cell density of fibroblast, ECs and epithelial cells [15,31]. Thickness of the CAM increases gradually during the early and mid phase of embryo growth due to the capillary migration from stroma region towards the inner shell membrane (ISM) [32]. The ISM lies very close to the basal lamina which is the outer surface of the CAM where gelatin sponges soaked with heparin are implanted. The thickness of CAM treated with heparin had increased indicating the proliferation and growth of the blood vessels and can be concluded that heparin has the potential to induce new vessels formation with maximum performance at 100 μM concentration within 72hours.

It is already known that the angiogenic response of CAM depends on diffusion of test materials through the CAM thereby indicating its involvement in angiogenesis. We studied the diffusion pattern of biotinylated heparin and found that heparin had diffused through various layers of CAM. The affinity of biotin with streptavidin has made it useful for numerous bioanalytical and biotechnological applications [33-35] using CAM and mouse embryo assays [36]. The diffusion pattern showed internalized action of heparin that could affect gene expression on endothelial cell and could stabilize, potentiate and transport growth factors which favor angiogenesis. This diffusion pattern was found to depend on the concentrations and implied the fact that though the higher concentration (150 μM) could favor new blood vessel formation up to certain extent might cause cellular damage. The diffusion of 100 μM heparin to the stroma region indicates that it would have a direct impact on angiogenesis and can favor sprouting of blood vessels.

## Conclusion

The present study evaluates the effects of HMWH on formation of capillary-like tubular structures using CAM assay. UFH of 15 kDa enhances its formation in contrast to LMWH which is known to decrease the formation of tubular endothelial structures. Thus the present *in ovo vivo* study has demonstrated that heparin possesses the angiogenesis inducing affinity and is significant at 100 μM concentration. These data provide a novel mechanism by which higher molecular weight heparins can promote formation of capillary-like tubular structures and might be of therapeutic significance in inducing angiogenisis during wound healing process while the low molecular weight constituents can be employed to inhibit angiogenesis as in tumor progression.

## Competing interests

The authors declare that they have no competing interests.

## Authors' contributions

Reji Bhuvanendran Rema carried out the experimental studies and made manuscript. Karthick Rajendran standardized CAM assay and helped in manuscript preparation and Malathi Ragunathan coordinated and helped in interpreting the data and reviewing the manuscript. All authors read and approve the final manuscript.

## References

[B1] Berry D Shriver Z Natke B Kwan CP Venkataraman G Sasisekharan R Heparan sulphate glycosaminoglycans derived from endothelial cells and smooth muscle cells differentially modulate fibroblast growth factor-2 biological activity through fibroblast growth factor receptor-1 Biochem J 2003 373 241 249 10.1042/BJ20021760 12659634 PMC1223466

[B2] Smorenburg SM Van Noorden CJ The complex effects of heparins on cancer progression and metastasis in experimental studies Pharmacol Rev 2001 53 93 105 11171940

[B3] Pacini S Gulisano M Vannucchi S Ruggiero M Poly-l-lysine/heparin stimulates angiogenesis in chick embryo chorioallantoic membrane Biochem Biophys Res Commun 2002 290 820 823 10.1006/bbrc.2001.6254 11785975

[B4] Zhang W Chuang YJ Swanson R Li J Seo K Leung L Lau LF Olson ST Antiangiogenic antithrombin down-regulates the expression of the proangiogenic heparan sulfate proteoglycan, perlecan, in endothelial cells Blood 2004 103 1185 1191 14563633 10.1182/blood-2003-08-2920

[B5] Sasaki T Larsson H Kreuger J Salmivirta M Claesson-Welsh L Lindahl U Hohenester E Timpl R Structural basis and potential role of heparin/heparan sulfate binding to the angiogenesis inhibitor endostatin EMBO J 1999 18 6240 6248 10.1093/emboj/18.22.6240 10562536 PMC1171687

[B6] Tiozzo R Cingi MR Croce MA Interaction of heparan sulfate and its fractions with endothelial cells in culture Int J Tissue React 1993 15 163 168 8188456

[B7] Nissen NN Shankar R Gamelli RL Singh A DiPietro LA Heparin and heparan sulphate protect basic fibroblast growth factor from non-enzymic glycosylation Biochem J 1999 338 Pt 3 637 642 10051433 PMC1220097

[B8] Taylor S Folkman J Protamine is an inhibitor of angiogenesis Nature 1982 297 1307 1912 10.1038/297307a0 6176876

[B9] Ribatti D Roncali L Nico B Bertossi M Effects of exogenous heparin on the vasculogenesis of the chorioallantoic membrane Acta Anat (Basel) 1987 130 257 263 10.1159/000146454 2449028

[B10] Pacini S Gulisano M Vannucchi S Ruggiero M Poly-L-lysine/Heparin Stimulates Angiogenesis in Chick Embryo Chorioallantoic Membrane Biochem Biophys Res Commun 2002 290 820 823 10.1006/bbrc.2001.6254 11785975

[B11] Ribatti D Chick embryo chorioallantoic membrane as a useful tool to study angiogenesis Int Rev Cell Mol Biol 2008 270 181 224 19081537 10.1016/S1937-6448(08)01405-6

[B12] Niemisto A Dunmire V Yli-Harja O Zhang W Shmulevich I Robust quantification of in vitro angiogenesis through image analysis IEEE Trans Med Imaging 2005 24 549 553 15822812 10.1109/tmi.2004.837339

[B13] Majumder S Rajaram M Muley A Reddy HEPARIN Tamilarasan KP Kolluru GK Sinha S Siamwala JH Gupta R Ilavarasan R Venkataraman S Sivakumar KC Anishetty S Kumar PG Chatterjee S Thalidomide attenuates nitric oxide-driven angiogenesis by interacting with soluble guanylyl cyclase Br J Pharmacol 2009 158 1720 1734 10.1111/j.1476-5381.2009.00446.x 19912234 PMC2801213

[B14] Gopi Krishna K Sinha S Majumder S Ajit M Siamwala JH Gupta R Chatterjee S Shear stress promotes nitric oxide production in endothelial cells by sub-cellular delocalization of eNOS: A basis for shear stress mediated angiogenesis Nitric Oxide 2010 22 304 315 10.1016/j.niox.2010.02.004 20188204

[B15] Yang EY Moses HE Transforming Growth Factor ß1- induced changes in cell Migration, Proliferation, and Angiogenesis in the chicken chorioallantoic membrane The J Cell Biology 1990 111 731 741 10.1083/jcb.111.2.731 PMC2116177 1696268

[B16] Reizis A Hammel I Ar A Regional and developmental variations of blood vessel morphometry in the chick embryo chorioallantoic membrane J Exp Biol 2005 208 2483 2488 10.1242/jeb.01662 15961734

[B17] Hillen F Griffioen AW Tumour vascularization: Sprouting angiogenesis and beyond Cancer Metastasis Rev 2007 26 489 502 10.1007/s10555-007-9094-7 17717633 PMC2797856

[B18] Ferrara N Gerber HP LeCouter J The biology of vegf and its receptors Nat Med 2003 9 669 676 10.1038/nm0603-669 12778165

[B19] Jain RK Molecular regulation of vessel maturation Nat Med 2003 9 685 693 10.1038/nm0603-685 12778167

[B20] Dudas J Ramadori G Knittel T Neubauer K Raddatz D Egedy K Kovalszky I Effect of heparin and liver heparan sulphate on interaction of HepG2-derived transcription factors and their cis-acting elements: altered potential of hepatocellular carcinoma heparan sulphate Biochem J 2000 350 Pt 1 245 251 10926850 PMC1221248

[B21] Parish CR The role of heparan sulphate in inflammation Nat Rev Immunol 2006 6 633 643 10.1038/nri1918 16917509

[B22] Risau W Ekblom P Production of a heparin-binding angiogenesis factor by the embryonic kidney J Cell Biol 1986 103 1101 1107 10.1083/jcb.103.3.1101 2427526 PMC2114278

[B23] Swinscode JC Carlson EC Capillary endothelial cells secrete a heparin-binding mitogen for pericytes J Cell Science 1992 103 453 461 1478947 10.1242/jcs.103.2.453

[B24] Mi-Sook L Eun-Joung Sae-Won L Myoung Sook K Kyu-Won K Yung-Jin K Angiogenic Activity of Pyruvic Acid inin Vivoandin VitroAngiogenesis Models Cancer Res 2001 61 3290 329311309282

[B25] Ribatti D Vacca A Giacchetta F Cesaretti S Anichini M Roncali L Damacco F Lipoprotein (a) induces angiogenesis on the chick embryo chorioallantoic membrane Eur J Clin Invest 1998 28 533 537 10.1046/j.1365-2362.1998.00322.x 9726032

[B26] Ribatti D Crivellato E Candussio L Nico B Vacca A Roncali L Dammacco F Mast cells and their secretory granules are angiogenic in the chick embryo chorioallantoic membrane Clin Exp Allergy 2001 31 602 608 10.1046/j.1365-2222.2001.00986.x 11359429

[B27] Rodriguez-Nieto S Gonzalez-Iriarte M Carmona R Munoz-Chapuli R Medina MA Quesada AR Antiangiogenic activity of aeroplysinin-1, a brominated compound isolated from a marine sponge FASEB J 2002 16 261 263 11772945 10.1096/fj.01-0427fje

[B28] Giannopoulou E Katsoris P Kardamakis D Papadimitriou E Amifostine inhibits angiogenesis in vivo J Pharmacol Exp Ther 2003 304 729 737 10.1124/jpet.102.042838 12538828

[B29] Matschke K Da Silva-Azevedo L Hlushchuk R Djonov V Baum O Annexins as cell-type-specific markers in the developing chicken chorionallantoic membrane Cell Tissue Res 2006 323 395 404 10.1007/s00441-005-0112-1 16344946

[B30] Balke M Neumann A Kersting C Agelopoulos K Gebert C Gosheger G Buerger H Hagedorn M Morphologic characterization of osteosarcoma growth on the chick chorioallantoic membrane BMC Res Notes 3 58 10.1186/1756-0500-3-58 PMC2838906 20202196

[B31] Wilting J Christ B Bokeloh M A modified chorioallantoic membrane (cam) assay for qualitative and quantitative study of growth factors Anat Embryol (Berl) 1991 183 259 271 2042751 10.1007/BF00192214

[B32] Schlatter P Konig MF Karlsson LM Burri PH Quantitative study of intussusceptive capillary growth in the chorioallantoic membrane (cam) of the chicken embryo Microvasc Res 1997 54 65 73 10.1006/mvre.1997.2022 9245646

[B33] Mark Howarth Daniel J-F Chinnapen Kimberly Gerrow Pieter C Dorrestein Melanie R Grandy Neil L Kelleher Alaa El-Husseini & Alice Y Ting A monovalent streptavidin with a single femtomolar biotin binding site Nature Methods 2006 3 4 267 273 10.1038/nmeth861 16554831 PMC2576293

[B34] Joshua R Wayment Joel MH Biotin-Avidin Binding Kinetics Measured by Single-Molecule Imaging Anal Chem 2009 81 336 342 10.1021/ac801818t 19117461

[B35] Zempleni J Wijeratne SS Hassan YI Biotin Biofactors 2009 35 36 46 10.1002/biof.8 19319844 PMC4757853

[B36] Fritzsch B Fast axonal diffusion of 3000 molecular weight dextran amines J Neurosci Methods 1993 50 95 103 10.1016/0165-0270(93)90060-5 7506342

